# Urine Microscopy: The Enduring Value of a Classic Test

**DOI:** 10.34067/KID.0000000985

**Published:** 2025-11-20

**Authors:** Corey Cavanaugh, Mark A. Perazella

**Affiliations:** 1 Department of Kidney Medicine, Cleveland Clinic Foundation, Cleveland, Ohio; 2 Department of Medicine, Section of Nephrology, Yale School of Medicine, New Haven, Connecticut

**Keywords:** AKI, clinical nephrology, mortality, renal tubular epithelial cells

Examination of the spun urine sediment has been available as a diagnostic tool since the 19th century. It is considered invaluable in the evaluation of various types of kidney disease, in particular AKI.^1^ Importantly, urine microscopy has been shown to provide valuable diagnostic and prognostic information in AKI. For example, it helps differentiate between various forms of AKI, predicts the severity and potential for recovery, and can guide clinical management.^2–4^ Unfortunately, through the years, urine microscopy has taken a diagnostic back seat with the push for centralized laboratory urine testing and automated urine technology. Many nephrologists have lost expertise or simply do not take the time collect, prepare, and view the spun urine sediment.

However, a rebirth of sorts has taken place with a surge in interest in and publication of several studies supporting the utility of urine microscopy in modern nephrology. In a 2020 study, serial urine microscopy was observed to enhance diagnostic findings of acute tubular injury that are initially missed by single urine examination.^5^ This suggests that repeat urine sediment examination should be pursued for persistent AKI not following a typical recovery course. Another study in 2022 showed that the presence of granular casts on urine microscopy despite fractional excretion sodium <1% in the setting of AKI is associated with biopsy-proven acute tubular injury, suggesting that low fractional excretion sodium is insufficient to exclude acute tubular injury.^6^ In a 2025 study, urine sediment examination combined with novel urine biomarkers was noted to improve the prediction of AKI progression and mortality in patients with sepsis-associated AKI.^7^ Thus, urine microscopy remains an important test for nephrologists and appears to provide utility in the ever-evolving approach to the definition, diagnosis, and management of AKI.

**Figure 1:**
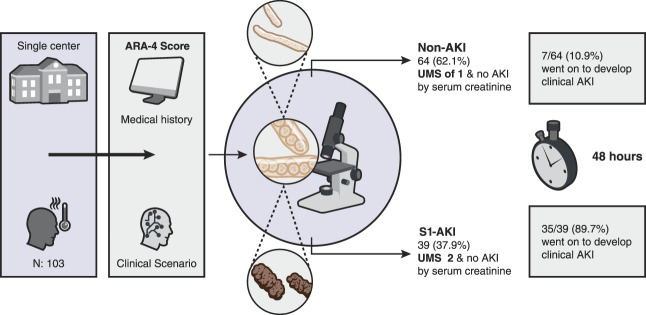
**Early identification and predictive value of AKI with urine microscopy with AKI risk assessment model score.** ARA-4, AKI Risk Assessment-4; S1-AKI, subclinical AKI; UMS, urine microscopy score.

To this point, in this issue of *Kidney360*,^8^ urine microscopy was used as a structural biomarker of tubular injury (in place of TIMP-2/IGFBP7) as part of the AKI Risk Assessment-4 (ARA-4) risk stratification model (Figure 1). The ARA-4 risk stratification model is a simple screening tool that is used to identify patients that are at risk to develop AKI.^9^ This relatively new predictive tool utilizes four simple criteria on the basis of clinical and laboratory data in an algorithm. These include (*1*) F1-Clinical scenario, (*2*) F2-Past history, (*3*) F3-Physical examination, and (*4*) F4-Laboratory analysis. The aim of this predictive tool is early identification of patients at moderate or high risk of AKI to prevent its progression and improve outcomes. Although this model has not been validated in prospective studies, it could have predictive potential, especially when powered by machine learning technology.

In this article, Claure-Del Granado and colleagues use the ARA-4 model to identify patients considered to be at moderate or high risk of AKI.^8^ To facilitate rapid screening, only the F1 and F2 criteria were used to identify at-risk patients. Of 432 hospitalized patients screened, 103 patients met criteria and were enrolled. In these patients, the Perazella urine microscopy score (UMS) on the basis of renal tubular epithelial cells and granular casts was calculated.^2^ Notably, trained laboratory technicians under the supervision of a nephrologist calculated the UMS scores, which were independently verified by a second nephrologist who was blinded to patient outcomes and not involved in the study. Furthermore, technicians received site-specific training in sediment identification and UMS scoring, including supervised practice and periodic competency assessments. On the basis of the UMS score, patients were separated into two groups (UMS ≥2 and UMS=1). Those with UMS ≥2 (*n*=39) were considered acute dialysis quality initiative subclinical AKI (stage S1-AKI), whereas UMS=1 (*n*=64) patients were classified as non-AKI. At 48 hours, 35/39 (89.7%) patients with S1-AKI and 7/64 (10.9%) non-AKI patients progressed to clinical AKI. Furthermore, 4/39 (10.3%) patients with S1-AKI versus 1/64 (1.6%) non-AKI patients required dialysis, whereas death occurred in 17/39 patients with S1-AKI versus 9/64 non-AKI patients. The statistical metrics were as follows: sensitivity=74.5%, specificity=92.9%, positive and negative predictive values=89.7 and 81.3%, positive and negative likelihood ratios=10.4 and 0.27, and area under the receiver operating curve=0.84.

The authors conclude that UMS offers promise as a structural biomarker for the early identification of subclinical AKI and prediction of its progression to clinical AKI, particularly when combined with AKI risk stratification tools like the AKI risk assessment model model. Furthermore, they note that its availability, simplicity, and reasonable diagnostic performance make it a practical option in both high-resource and resource-constrained settings if validated in other studies. These appear to be reasonable interpretations of their study. However, there are notable limitations to this study. It is a small, single-center observational study of only non-intensive care unit hospitalized patients. As with other studies without formal certification programs, clinical application of UMS scores may vary, and the potential for interobserver variability in urine sediment interpretation may affect the UMS scoring reproducibility. The authors attempted to reduce these constraints by training (and retraining) nephrology fellows and technicians in UMS scoring using standardized protocols.

How should clinicians and researchers view these data, and importantly, should these results be applied to our current approach to AKI and subclinical AKI as defined by the acute dialysis quality initiative group?^10^ Urine microscopy has been previously shown to be useful for AKI diagnosis and prognosis while also performing at least as good as novel biomarkers. In this study, urine microscopy was able to identify subclinical AKI in a group of patients considered to be at moderate or high risk on the basis of the ARA-4 screening tool. This seems meritorious, but it is unclear whether identifying subclinical AKI (lack of serum creatinine or urine output criteria for AKI) is amenable to interventions that prevent or reduce progression to clinical AKI and change clinical outcomes. Also, there remain issues of concern for urine microscopy, such as lack of widespread competence in sediment examination, variable interobserver reliability, and time constraints for busy clinicians. The first two issues can be potentially overcome with increased training during nephrology fellowship, whereas the last one is hard to address. Regarding the utility of the ARA-4 risk stratification model, this needs to be determined with additional study. This predictive tool has not been widely tested or validated in prospective studies, and most clinicians are unfamiliar with it. Furthermore, will this tool offer any benefits (ease and accuracy) over the currently available AKI prediction models or the growing number of novel biomarkers (alone or as panels) that reflect renal tubular epithelial cell stress, injury, or inflammation?
